# The role of PCBP1 in carbon ion-induced ferroptosis and inhibition of lung adenocarcinoma proliferation

**DOI:** 10.3389/fpubh.2024.1496439

**Published:** 2025-01-08

**Authors:** Zhenchao Kang, Zihan Fu, Xuejiao Tian, Yichao Geng, Qiuning Zhang, Yanli Liu, Xinhua Wang, Hongtao Luo, Zhen Yang

**Affiliations:** ^1^Department of Radiotherapy, Liangzhou Hospital of Wuwei City, Wuwei, China; ^2^School of Public Health, Gansu University of Chinese Medicine, Lanzhou, China; ^3^Institute of Modern Physics, Chinese Academy of Sciences, Lanzhou, China; ^4^Tumor Hospital of Gansu Province, Lanzhou, China; ^5^Gansu Provincial Hospital of TCM, Lanzhou, China

**Keywords:** carbon ion, ferroptosis, PCBP1, lung adenocarcinoma, proliferation

## Abstract

**Objective:**

To investigate the role of PCBP1 in the inhibition of lung adenocarcinoma proliferation by carbon irradiation.

**Methods:**

A549 cells were irradiated with different doses of carbon ions to observe clonal survival and detect changes in cell proliferation. Whole transcriptome sequencing and the Illumina platform were used to analyze the differentially expressed genes in A549 cells after carbon ion irradiation. The relationship between the expression levels of PCBP1, ACSL4, and ALOX15 and survival was analyzed by combining data from the UCSC database and the Kaplan–Meier Plotter public platform. Additionally, the knockdown of the poly (rC)-binding protein 1 (PCBP1) gene using siRNA techniques was employed to further investigate the relationship between the expression levels of PCBP1 and ALOX15. To investigate the relationship between ALOX15 expression and survival, we assessed changes in key indicators of ferroptosis (mitochondrial morphology, ROS, MDA, and divalent iron) in A549 cells after knocking down the PCBP1 gene using siRNA technology. Additionally, the expressions of PCBP1, ACSL4, and ALOX15 in different groups were further analyzed through RT-PCR and Western blot techniques. The differential expression of PCBP1, ACSL4, and ALOX15 in NSCLC tissues was found to correlate with clinical prognosis for survival.

**Results:**

Carbon ions significantly inhibited the proliferation of A549 cells, and 5.16 Gy carbon ions significantly induced the expression of differentially expressed genes in these cells. Additionally, carbon ions inhibited the expression of PCBP1, which led to alterations in mitochondrial morphology in lung adenocarcinoma cells. This was associated with a significant increase in the levels of ROS, MDA, and Fe^2+^. Furthermore, low expression of PCBP1 promoted ferroptosis by increasing the expression of ACSL4 and ALOX15.

**Conclusion:**

Carbon ions decreased the expression of PCBP1 in A549 cells, and low expression of PCBP1 inhibited tumor proliferation by promoting ferroptosis.

## 1 Introduction

Non-small cell lung cancer (NSCLC) is the most predominant pathological type, accounting for about 80–85% of all lung cancer cases. Despite continuous improvements in therapeutic methods and technology, the clinical efficacy of treatments has improved; however, the 5-year survival rate for advanced NSCLC remains (1), and new therapeutic options need to be researched urgently. The clinical application of high LET rays in NSCLC reflects the advantages of a high local control rate, low critical organ exposure, and a low rate of adverse effects. Arachidonate-15-Lipoxygenase (ALOX15) plays a central role in lipid peroxidation and ferroptosis (2), and carbon ion rays lead to DNA double-stranded damage in tumor cells, as well as inducing changes in tumor metabolism factors, which damage nucleic acids, proteins and lipids and cause cellular damage and even death (3). Failure of the iron and RNA-binding activity of PCBP1 and inhibition of PCBP1 allows cells to perceive a lack of cellular iron and leads to an inadequate supply of iron to syncytia that require iron to function properly (4, 5).It has been shown that PCBP1 plays an important role in ferritin phagocytosis-mediated iron mutations in head and neck cancer cells, and PCBP1 also regulates the translation of ALOX15 by binding to the 3′-UTR regional regulatory complex that controls the assembly of 80s ribosomal subunits (6). Ferroptosis is a novel iron-dependent mode of programmed cell death induced by the excessive accumulation of peroxidized lipids. Radiotherapy induces the ionization and decomposition of water in cancer cells, generating excess reactive oxygen species (ROS), which promote lipid peroxidation (7). Long-chain acyl-CoA synthetase 4 (ACSL4) is a key molecule in ferroptosis, and radiation upregulates ACSL4 expression to promote ferroptosis. However, it remains unknown whether carbon ions can induce other modes of cell death and what role these modes may play in radiotherapy resistance. Therefore, it is particularly important to explore the potential mechanisms of ferroptosis in carbon ion radiation.

## 2 Methods

### 2.1 Cell culture and transfections

A549 cells were cultured in RPMI1640 medium supplemented with 10% fetal bovine serum and penicillin–streptomycin solution (penicillin 100 U/mL, streptomycin 100 mg/mL) in an environment of 5% carbon dioxide/95% air at a humidity of 37°C. The medium was changed daily and the cells were in the logarithmic growth phase at the time of the experiment (1 × 10^7^cells). PCBP1-AS1 small interfering RNA (si-PCBP1-AS1) and corresponding control (si-NC) were purchased from RiboBio. A549 cells were transfected with oligomers and plasmids using Lipofectamine 3000 (2067450) reagent as provided by the manufacturer. A549 cells were introduced into cells with 50 nM siRNA oligo or 2 μg overexpression plasmid at approximately 70% cell density. Untreated cells were set as blank control group and transfected with empty vector and NC-siRNA as negative control. The efficiency of knockdown and overexpression was detected by qRT-PCR and fluorescence microscopy at 24 h post-transfection. Subsequent experiments were performed 48 h after transfection.

### 2.2 Irradiation conditions

Heavy ions were obtained by irradiating the cells with a carbon ion (^12^C^6+^) beam from the Deep Therapeutic Terminal of the Institute of Modern Physics, Chinese Academy of Sciences (HIRFL-CSR). (Ray parameters: 100 MeV energy, 1 Gy/min dose rate, 5 mm wide Bragg peak, 5 cm*5 cm radiation field) to irradiate the cells. The irradiation doses were 0, 2, 4, and 6 Gy.

### 2.3 Cell proliferation

EdU assay was performed using an EdU assay kit (RiboBio, China). In short, cells were harvested after irradiation with carbon ions 5.16Gy and re-inoculated into 48 h 96-well plates (1 × 10^4^ cells/well) for EdU assay. After incubated with EdU (50 μM) for 2 h, the samples were fixed in 4% formaldehyde for 15 min. Subsequently, cells were permeabilized with 0.3% TritonX-100 for 10 min and then incubated with glycine for 5 min. Cell nuclei were stained with Hoechst 33342 for 5 min at room temperature. The EdU-treated cells were imaged and counted under an Olympus FSX100 microscope.

### 2.4 *RNA* extraction, library construction, and RNA-seq data processing and analysis

Total RNA was extracted from carbon ion irradiated cells using Trizol reagent reagent, cDNA was synthesized using mRNA as template. Second strand synthesis and deletion of mRNA produced double stranded cDNA (dscDNA), purification of repaired cDNA ligated to the junction dscDNA, PCR enriched library, PCR amplification, purification, and library QC. Using 2 × 150 bp paired-end sequencing on Illumina Novaseq™ 6,000, with fold difference (fold change) ≥2 (log2FC absolute value ≥1) as the change threshold and q < 0.01 (q value is the corrected *p* value) as the criterion to screen for differentially expressed genes (|log2FC| ≥1.5 & q < 0.01), in the set of comparison groups, the results of differentially expressed genes, differentially expressed gene enrichment analysis, GSEA analysis, and differential variable shear were obtained.

### 2.5 Database were downloaded for gene expression analyses and survival analyses

Data were downloaded from UCSC^1^ and the HPA^2^ database to analyze the expression of related regulatory genes in NSCLC. The prognostic value of related molecules in NSCLC patients was analyzed using the Kaplan–Meier Plotter online tool.^3^ A *p*-value of <0.05 was considered statistically significant.

### 2.6 Transmission electron microscopy

Mitochondrial morphology was observed using transmission electron microscopy (TEM). A549 cells were collected and fixed at 4°C, embedded in 1% agarose, and then fixed with 1% osmotic acid in the dark at room temperature for 2 h. Gradient dehydration was performed in 30, 50, 70, 80, 90, and 100% acetone for 15 min each. Then, a resin-based gradient embedding was performed at 37°C followed by 65°C overnight. Polymerization, sectioning and staining were observed using a Hitachi TEM system and images were captured for analysis.

### 2.7 Measurement of intracellular ROS levels

Intracellular ROS levels were measured using a ROS detection kit (Biyuntian, China). Cells were inoculated into 96-well plates as described above, treated with 5.16Gy irradiation of carbon ions, and then incubated with DCFH-DA at 37°C for 20 min. Then, labeled A549 cells were trypsinized and analyzed by flow cytometry.

### 2.8 Cell proliferation

Cell proliferation was evaluated using the Cell counting kit (CCK-8) (Biyuntian, China). The cells were incubated at 37°C for 24, 48, 72, and 96 h. Subsequently, the medium was discarded then the chromogenic solution at 10:1 was prepared. For incubation, a color-developing solution (10 μL) was added to the 96 well plates at 37°C for 2 h. The optical density (OD) was detected using the UV spectrophotometer at 450 nm.

### 2.9 Measurement of monodehydroascorbate content

Lipid peroxidation was detected using the MDA kit (Biyuntian, China) according to the manufacturer’s instructions. A 100 μL sample of cell lysate was placed in 1.5 mL of reaction buffer and 1.4 mL of 0.2 M Tris-0.16 M KCl (pH 7.4) and incubated at 37°C for 30 min, followed by the addition of 1.5 mL of thiobarbituric acid reagent. The mixture was then heated in a boiling water bath for 10 min. After ice-cooling, 3.0 mL of pyridine:n-butanol (3,1, v/v) and 1.0 mL of 1 M NaOH were added and mixed by shaking. The absorbance was read at 548 nm. Protein concentration in cell lysates was determined using a protein assay kit (Thermo Fisher). MDA levels were expressed as nmol/g protein. Three parallel experiments were performed and the results were expressed as mean values.

### 2.10 Intracellular ferrous assay

The Intracellular Ferrous Colorimetric Assay Kit was used to determine intracellular Fe^2+^ levels. Approximately 1 × 10^6^ cells were extracted and homogenized with 200 μL of lysis buffer on ice for 10 min, and then centrifuged at 15,000 × g for 10 min to collect the supernatant. Then 80 μL of supernatant was treated with iron control reagent for 10 min at 37°C. Absorbance at 593 nm was measured by an enzyme marker and analyses by manipulation according to the ferrous ion detection kit (E-BC-K881-M) provided by Elabscience.

### 2.11 Confocal immunofluorescence

Confocal immunofluorescence microscopy was performed on A549 cells. Briefly, cells were fixed and incubated with rabbit polyclonal antibody anti-PCBP1 antibody at 4 °C overnight and then incubated with secondary antibodies (ImmunoWay) for 1 h. Finally, the cells were incubated with DAPI for 5 min and viewed with a Fluoview FV1000 microscope (Olympus).

### 2.12 RNA extraction and quantitative PCR

Total RNA was extracted from the cells using TRIzol reagent and RNA concentration was measured using a Qubit 8,000 (Thermo Fisher). qRT-PCR was performed on an ABI7500 system (AppliedBiosystems, CA) using Hieff^®^ qPCR SYBR^®^ Green Master Mix. cycling parameters were as follows: initial denaturation at 95°C for 60 s, 95°C for 10 s, 58°C for 20 s, and 72°C for 20 s. 40 cycles were performed: PCBP1-F: 5′- CAGAGGTGAAA GGCTATTGG-3′, R: 5′- GGCAGCAGAGCCAGTGATAG-3′, ACSL4-F:5′-CATCCCTGGAGCAGATACTCT-3′, R:5′- TCACTTAGGATTTCCCTGGTCC3′, ALOX15-F:5′-GGGCAAGGAGACAGAACTCAA-3′, R:5-CAGCGGTAACAAGGGAACCT-3′, GAPDH-F: 5′-CTCCTCCACCTTTGACGCTG-3′, R:5′- TCCTCTTGTGCTCTCTTGCTGG-3′. GAPDH was used as the control, and the processing was calculated by the 2^-ΔΔCt^ method based on the Ct data obtained from the assay, ΔΔCt = ΔCt of the experimental group − ΔCt of the control group, and ΔΔCt = the difference between the target gene Ct of the experimental group − the internal reference Ct of the experimental group.

### 2.13 Western blotting analysis

Total protein was extracted with RIPA lysis buffer, separated by 10% SDS-PAGE, and then transferred to PVDF membrane. Antibodies were used for immunoblotting: polyclonal ALOX15 (Cat. No. bs-34007R), PCBP1, ACSL4, *β*-ACTIN antibody and horseradish peroxidase (HRP)-conjugated secondary antibody (anti -rabbit antibody) were purchased from Proteintech (No. 14523-1-AP, No.22401-1- Ig, No.66009-1-Ig, Cat.No.66009-1-AP).

### 2.14 Statistical analysis

Data are expressed as mean ± standard deviation (^−^x ± sd) and were statistically analyzed using GraphPad Prism 7. Student’s *t*-test was used for comparison between two groups, and statistical significance was assessed by one-way analysis of variance (ANOVA) test for comparison between multiple groups, with *p* < 0.05 defined as statistically significant, *p < 0.05, **p < 0.01.

## 3 Results

### 3.1 Carbon ions inhibit A549 cell clonal survival and cell proliferation

**Figure 1:**
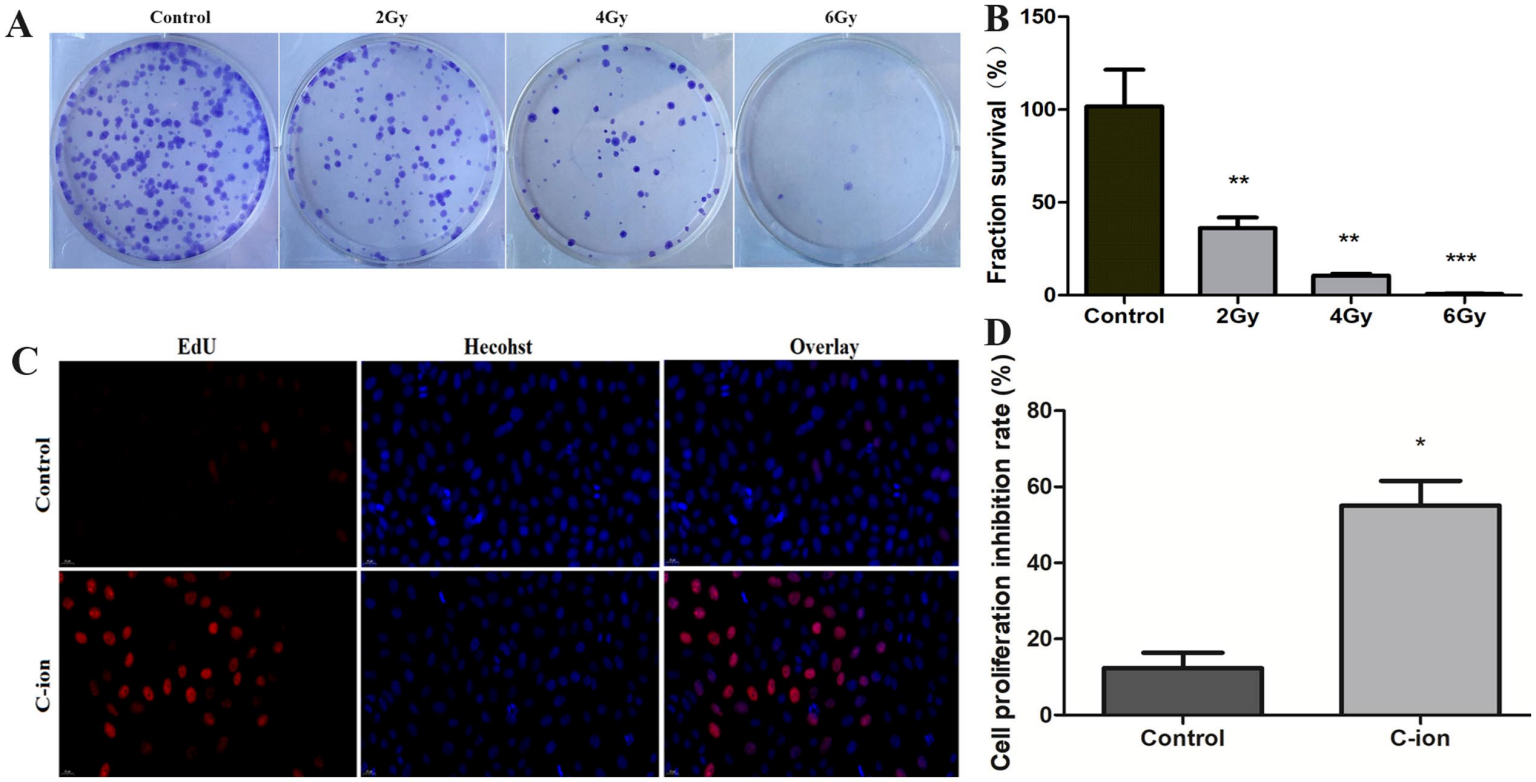
Changes in clonogenic survival fraction and cell proliferation of carbon ion irradiated cells. **(A)** Clonogenic survival of A549 cells. **(B)** Calculation of clonogenic survival fraction of A549 cells. **(C)** Results of EdU staining after irradiation. **(D)** Changes in cell proliferation rate. *p < 0.05, **p < 0.01, and ***p < 0.001 compared to controls.

After different doses of carbon ion irradiation, the clonal survival of A549 cells was observed (Figure 1A), compared with the control group, the number of cell clonal survival was significantly reduced after 2Gy carbon ion irradiation, and this change was even more significant after 4Gy irradiation (Figure 1B), with a clonal survival fraction of 10%, and at an irradiation dose up to 6Gy, the cell clonal survival fraction was 0.6%. Further, we chose 5.16Gy irradiation of A549 cells to detect the changes in cell proliferation (Figure 1C), compared with the control group, the cell proliferation ability was weakened after carbon ion irradiation, and the cell proliferation ability was significantly reduced (Figure 1D).

### 3.2 Analysis of transcriptome differentially expressed genes by carbon ion irradiation

**Figure 2:**
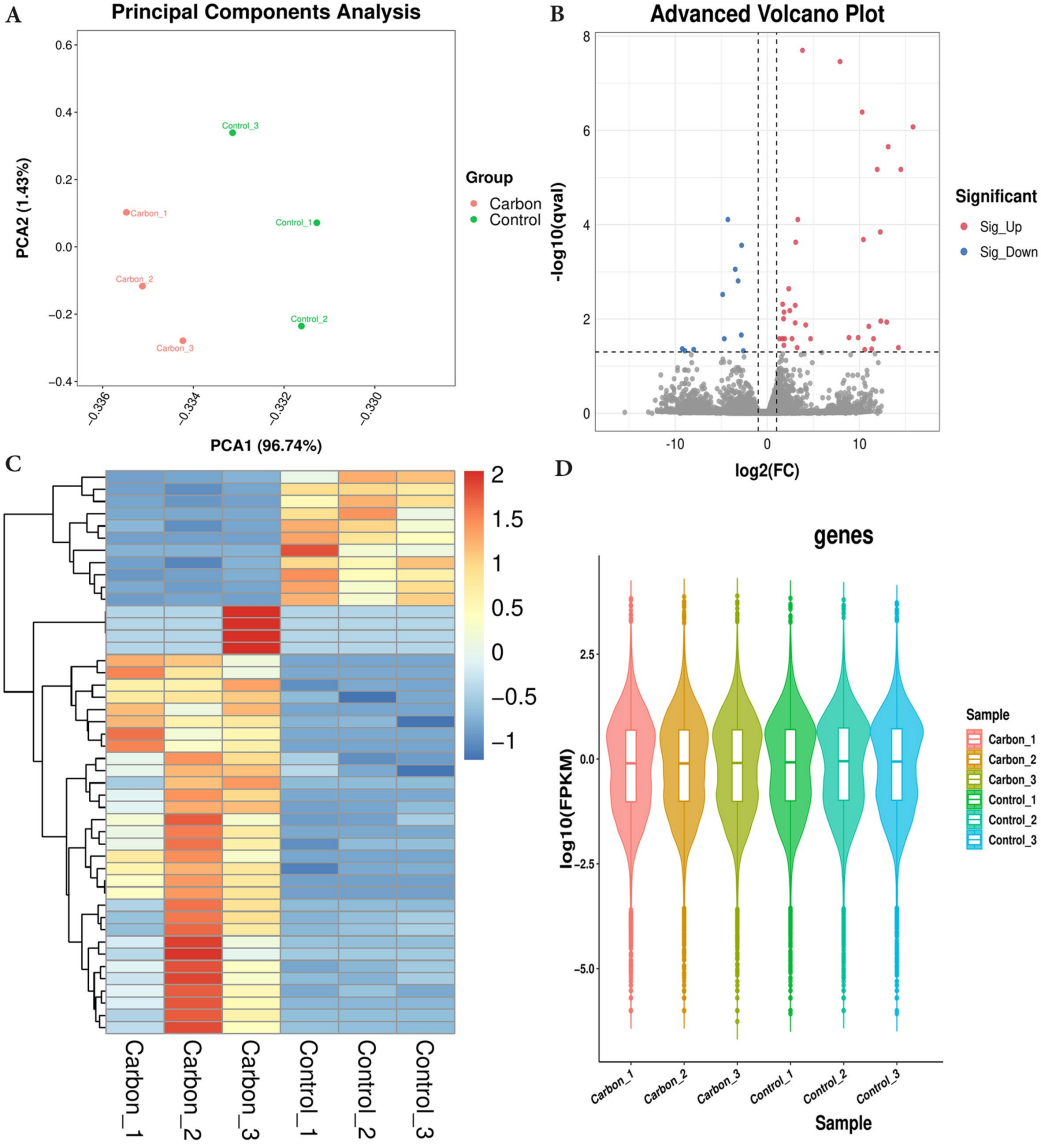
Differentially expressed genes were filtered out by gene-set analysis. **(A)** Principal component analysis (PCA) analysis of transcriptome sequencing samples. **(B)** Volcano plot analysis. **(C)** Heatmap cluster of DEGs. **(D)** Violin map of FPKM values.

PCA analysis was performed on all samples, and the results of PCA analysis are shown in Figure 2A. Control and carbon ion transcriptomes group principal component analysis with good reproducibility. On the basis of RNA-seq analysis, the DEGs were screened out after irradiation by carbon ions, of which 46 genes fit into the range of |log2FC| ≥ 1 & q < 0.05, and the number of genes with up-regulated 5.16Gy carbon ion expression at this threshold was 15 and the number of down-regulated genes was 31 (Figure 2B). Clustering and difference results of sequencing samples suggested that there were significant differences between Control and Carbon groups (*n* = 3) in this study, and clustering based on the samples resulted in higher similarity of data points within the same group, and lower results of similarity of data points between the same groups (Figure 2C). We listed the information of some of the differentially expressed genes (Table 1), there were significantly down-regulated expression of SLC25A36, SP3, PCBP1, and ATM, while ISG15, STAT6, CCL5, and RPL29P11 were up-regulated. These plots revealed similar gene expression values across the samples, indicating the absence of biological replicate samples. The sample has symmetry and the distribution is well dispersed (Figure 2D).

**Table 1:** Differentially expressed genes in A549 cells after carbon ion irradiation.

Gene_ID	Name	FC	Log2(FC)	pval	Regulation
ENSG00000114120	SLC25A36	0.42	−1.25	0.00	Down
ENSG00000172845	SP3	0.42	−1.26	0.00	Down
ENSG00000169564	PCBP1	0.33	−0.17	0.00	Down
ENSG00000187608	ISG15	3.36	1.75	0.00	Up
ENSG00000149311	ATM	0.40	−1.32	0.00	Down
ENSG00000101972	STAG2	0.38	−1.38	0.00	Down
ENSG00000011405	PIK3C2A	0.42	−1.24	0.00	Down
ENSG00000163660	CCNL1	0.42	−1.24	0.00	Down
ENSG00000166888	STAT6	1.42	0.51	0.00	Up
ENSG00000123473	STIL	0.46	−1.13	0.00	Down
ENSG00000170759	KIF5B	0.46	−1.12	0.00	Down
ENSG00000169251	NMD3	0.48	−1.06	0.00	Down
ENSG00000115966	ATF2	0.46	−1.12	0.00	Down
ENSG00000144895	EIF2A	0.45	−1.16	0.00	Down
ENSG00000165732	DDX21	0.52	−0.94	0.00	Down
ENSG00000271503	CCL5	2.87	1.52	0.00	Up
ENSG00000135913	USP37	0.42	−1.27	0.00	Down
ENSG00000104408	EIF3E	0.51	−0.97	0.00	Down
ENSG00000113658	SMAD5	0.47	−1.09	0.00	Down
ENSG00000113594	LIFR	0.43	−1.23	0.00	Down
ENSG00000126777	KTN1	0.45	−1.16	0.00	Down
ENSG00000071794	HLTF	0.44	−1.17	0.00	Down
ENSG00000224858	RPL29P11	3.09	1.63	0.00	Up

FC, fold change; pval, corrected p.

### 3.3 GO enrichment analysis and KEGG pathway analysis

**Figure 3:**
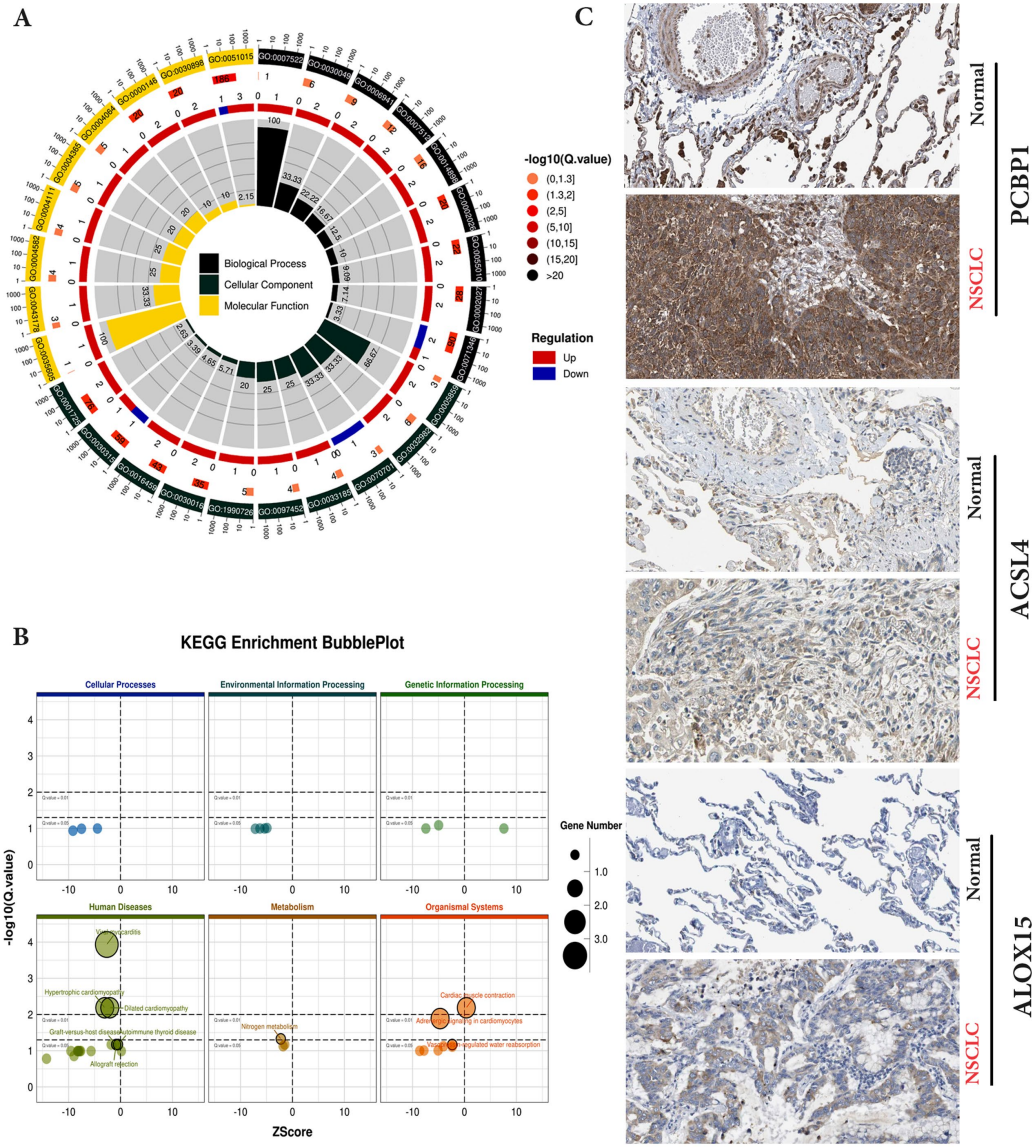
KEGG pathway enrichment and GO analysis of differential genes in control and irradiation by transcriptome sequencing. **(A)** Differential gene GO enrichment analysis. **(B)** Differential gene KEGG enrichment analysis. **(C)** Validation of protein expression levels of hub genes in the HPA database.

The GO CC analysis revealed that the differentially expressed molecules were involved in the nucleus, nucleoplasm, cytoskeleton and cytoplasm, etc. (Figure 3A). Nucleus, nucleoplasm, cytoskeleton and cytoplasm (Figure 3A). In order to further investigate the molecular regulatory pathways mainly affected by carbon irradiation, KEGG pathway enrichment analysis was performed on the RNA-seq data. The results showed that the main response-stimulating molecular pathways after carbon ion irradiation were homologous recombination repair, cytoplasmic DNA sensing pathway, metabolism and adrenergic signaling in cardiomyocytes (Figure 3B). PCBP1 is a cytosolic iron chaperone involved in the delivery of iron to ferritin and its depletion in human cells is known to inhibit ferritin iron loading, leading to increased cytosolic iron pools. PCBP1 is involved in iron metabolism. To verify the enhanced expression of PCBP1, ACSL4 and ALOX15 in NSCLC and normal, we accessed IHC data from the HPA database. Moreover, the HPA database verified that PCBP1, ACSL4 and ALOX15 protein expression levels and mRNA expression were consistent (Figure 3C).

### 3.4 Differential expression of PCBP1 and ACSL4, ALOX15 in NSCLC

**Figure 4:**
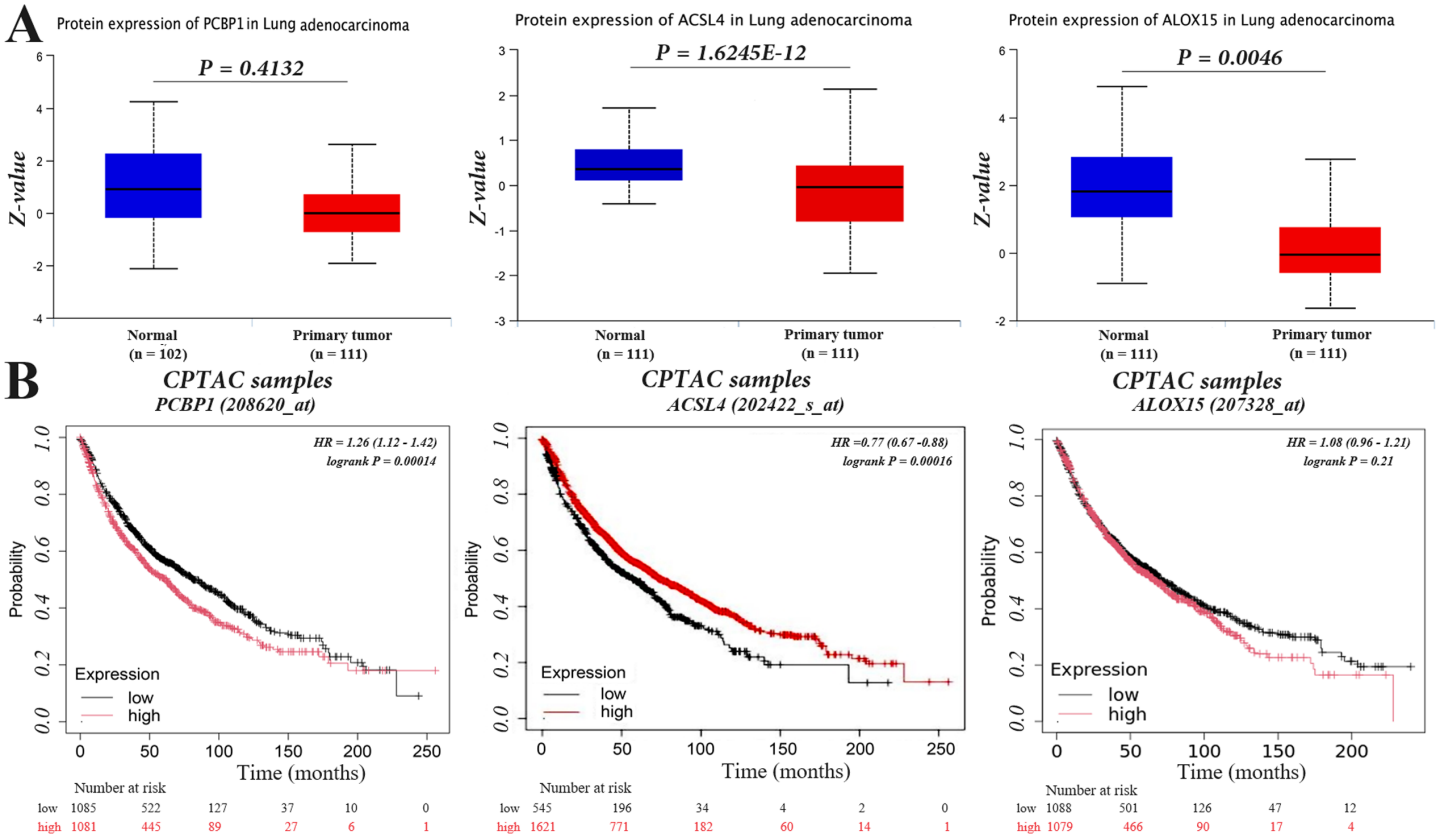
PCBP1, ACSL4, and ALOX15 expression in NSCLC and overall survival. **(A)** Differentially expressed. **(B)** Survival curves.

We chose PCBP1 as the main study gene, and analyzed and compared the relationship between the expression differences of PCBP1, ACSL4 and ALOX15 and the survival of NSCLC patients from the UCSC (see text footnote 1) database. The results suggested that the PCBP1 gene was highly expressed in NSCLC tumor tissues and lowly expressed in lung normal tissues, with no statistically significant difference between the two (*p* > 0.056), whereas ACSL4 and ALOX15, which were lowly expressed in NSCLC tumor tissues and highly expressed in lung normal tissues (Figure 4A), were both significantly different compared to the other (*p* < 0.01).

To investigate whether the expression of PCBP1, ACSL4, and ALOX15 in NSCLC could predict patient survival, we analyzed the overall survival (OS) of NSCLC patients in high and low expression groups of these molecules using the Kaplan–Meier Plotter database. The results indicated that the expression level of PCBP1 was significantly negatively correlated with survival (*p* < 0.05), with longer OS observed in the low expression group. In contrast, high expression of ACSL4 was associated with prolonged survival in NSCLC patients (Figure 4B) (*p* < 0.05). However, there was no significant difference in survival time between the high and low expression groups for ALOX15 (*p* > 0.05).

### 3.5 Carbon ions mediate mitochondrial and ROS alterations via PCBP1

**Figure 5:**
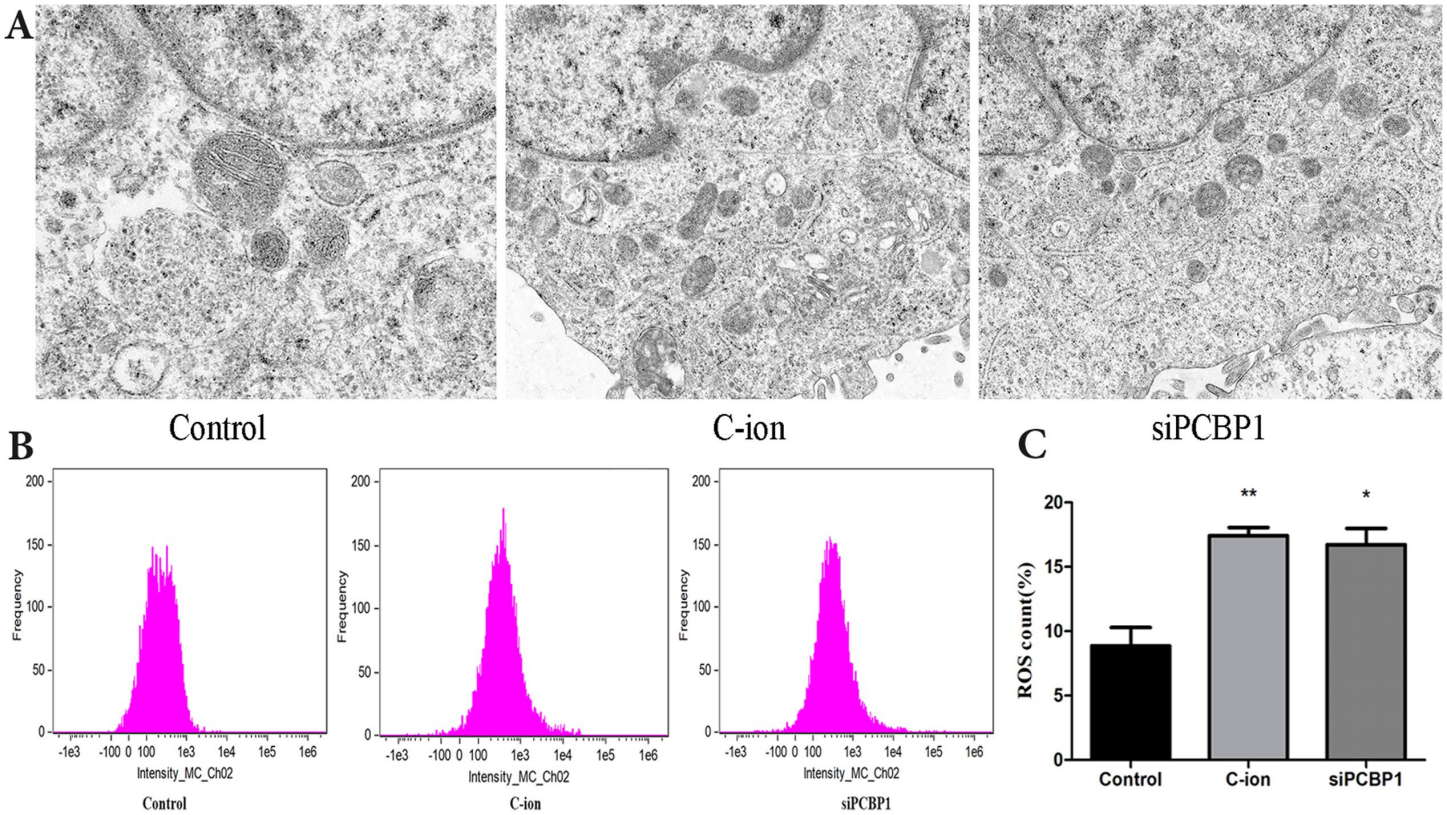
Effects of carbon ion radiation and *siPCBP1* on mitochondrial morphology and ROS in A549 cells. **(A)** Electron microscopy of the mitochondrial structure of the A549 cells in each group. **(B)** Reactive oxygen species generation was detected through flow cytometry. **(C)** ROS level quantification. *p < 0.05, **p < 0.01 compared to controls.

Furthermore, we knocked down PCBP1 in A549 cells using siRNA technology and observed changes in mitochondrial morphology and ROS levels simultaneously with 5.16 Gy carbon ion irradiation in the A549 cell group. The results showed that the knockdown of PCBP1 and carbon ion irradiation significantly altered the morphology of A549 mitochondria, resulting in the rupture of the outer mitochondrial membrane, volume reduction, and cristae reduction (Figure 5A). The reduction in ROS level observed may result from decreased ROS generation or increased antioxidant capacity. There were also significant changes in the level of ROS (Figure 5B). Compared to the control group, there was a significant increase in ROS levels in the carbon irradiation and *siPCBP1* group (Figure 5C) (*p* < 0.05), with no significant difference between the two experimental groups (*p* > 0.05).

### 3.6 Inhibiting the expression of PCBP1 to increase MDA and ferric ions (Fe^2+^)

**Figure 6:**
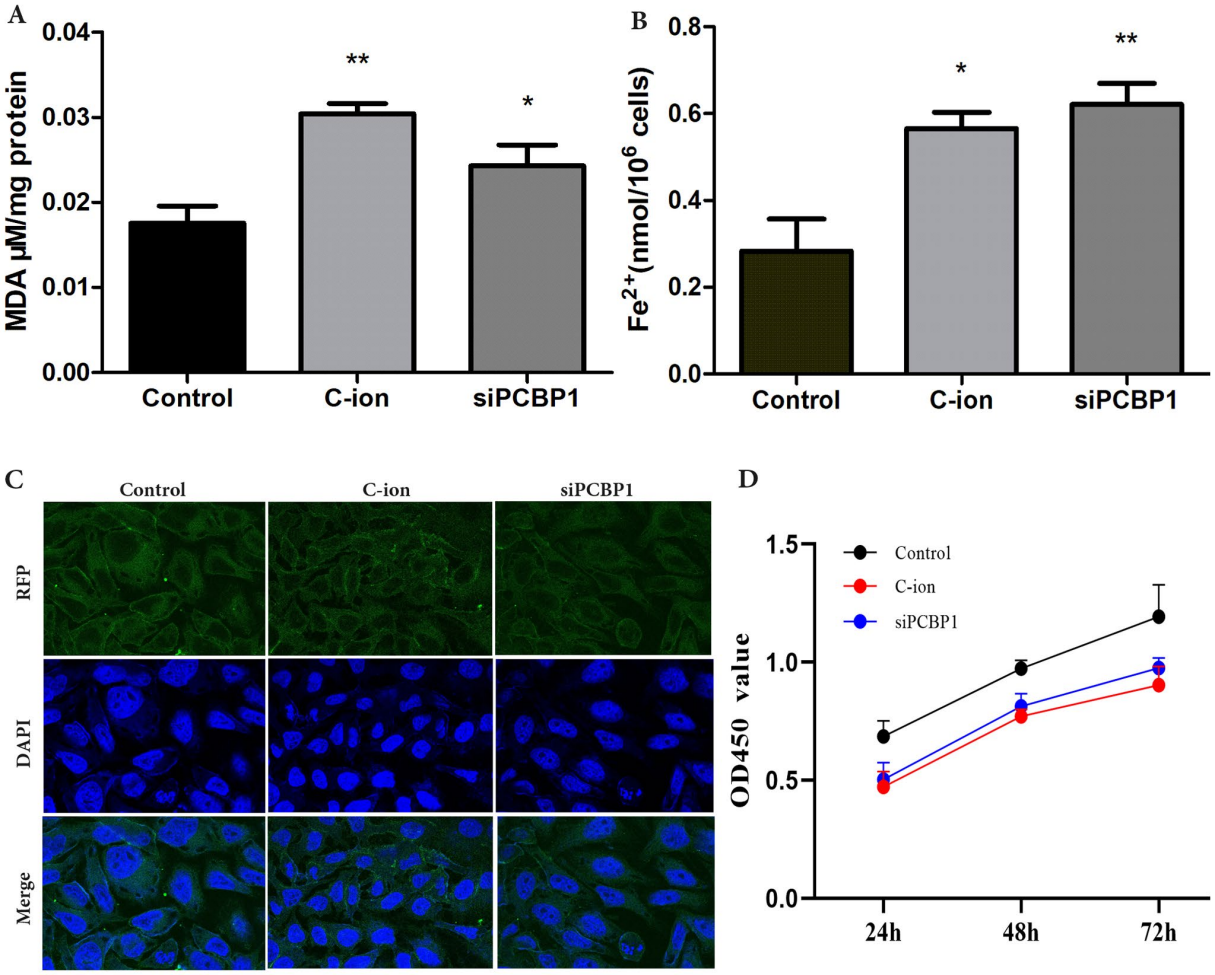
Carbon ion irradiation and knockdown of PCBP1 promote MDA and ferrous ion concentrations in cells. **(A)** MDA content. **(B)** Ferrous irons content. **(C)** Representative confocal microscopy of DAPI stained for PCBP1. **(D)** CCK-8 assay was performed after siRNA transfection. *p < 0.05, **p < 0.01 compared to controls.

The content of the lipid peroxidation product MDA was enhanced in A549 cells after carbon ion irradiation. Further experiments revealed that we examined the content of MDA and Fe^2+^ in the cells, finding a significant increase in MDA levels after both carbon ion irradiation and the knockdown of PCBP1 expression (Figure 6A) (*p* < 0.05). Detection of Fe^2+^ content in the cells across each group showed that the accumulation of free iron in A549 cells increased following carbon ion irradiation and PCBP1 knockdown. Notably, the amount of Fe^2+^ in the *siPCBP1* group was significantly higher compared to the control group (Figure 6B) (*p* < 0.01). We further confirmed these results by confocal studies, showing that PCBP1 colocalized in A549 (Figure 5C). Carbon ion radiation OR low expression of PCBP1 inhibits A549 cell proliferation (Figure 5D).

### 3.7 PCBP1 regulates the expression of ferroptosis related proteins in A549 cells

**Figure 7:**
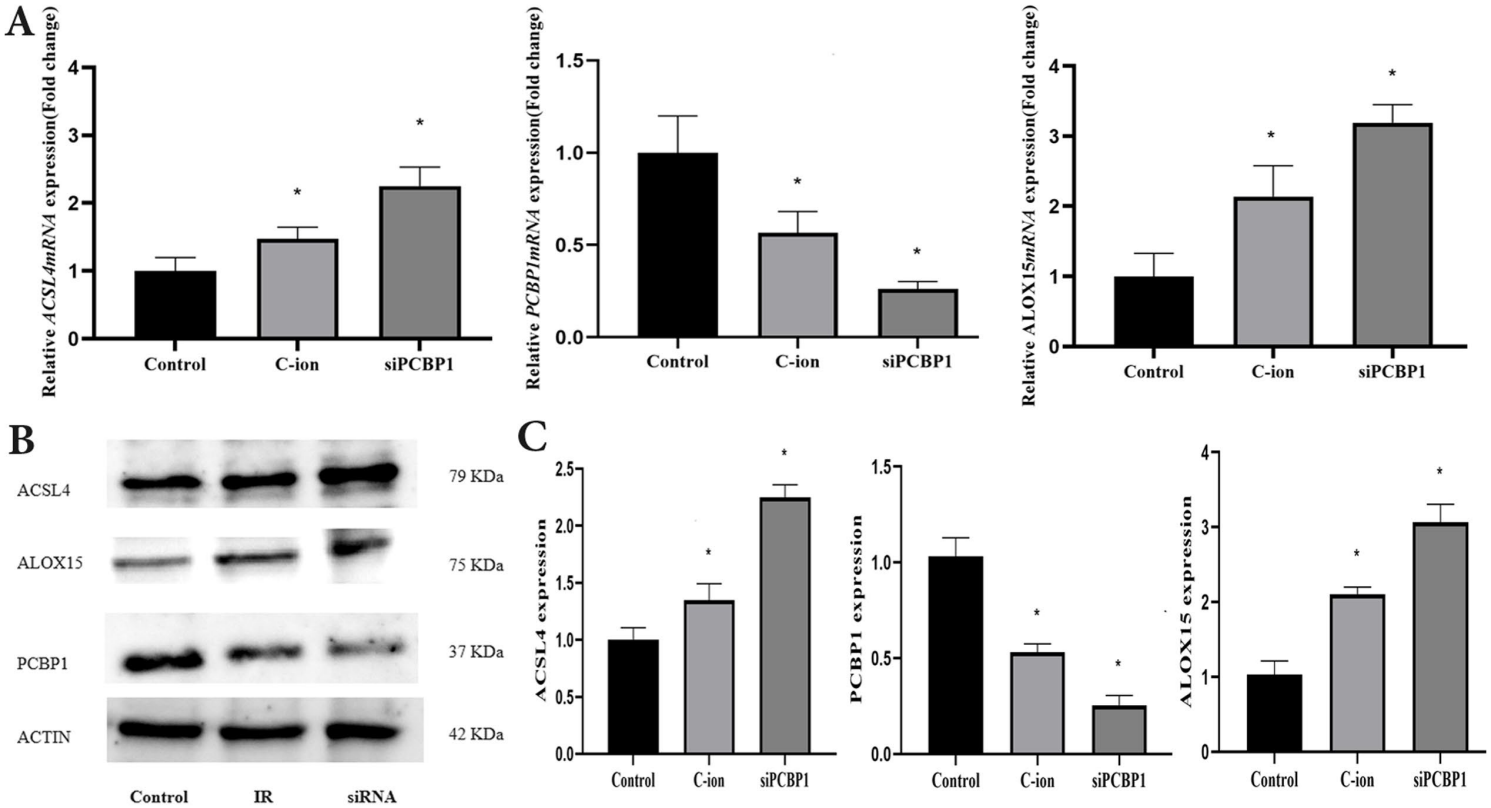
Changes in the expression levels of ferroptosis related molecules in A549 cells after carbon ions and knockdown of PCBP1. **(A)** The relative mRNA expression. **(B)** Representative Western blot. **(C)** Western blot analysis. *p < 0.05 compared to controls.

To further explore the role of PCBP1 in ferroptosis, the expression of PCBP1, ACSL4, and ALOX15 was analyzed using RT-PCR and immunoblotting in carbon ion-irradiated and siPCBP1 groups (Figure 7). The results showed that 5.16 Gy carbon ion irradiation significantly upregulated the expression of ACSL4 and ALOX15 at both transcriptional and translational levels in A549 cells (Figure 7A) (*p* < 0.05), while downregulating the expression of PCBP1 (Figure 7B). A significant increase in both transcriptional and translational levels was also observed in the knockdown of PCBP1, along with a significant increase in ACSL4 and ALOX15 (Figure 7C) (*p* < 0.05).

## 4 Discussion

Carbon ions have unique biological and physical advantages and are considered an effective treatment for cancer (8), which significantly inhibits cell proliferation and promotes apoptosis and DNA double-strand breaks. Radiation not only induces DNA damage, lipid peroxidation and protein changes (9), but also affects lipid changes in polyunsaturated fatty acids (PUFAs) and phospholipids that constitute cell membranes. The research group confirmed that carbon ions altered lipid peroxidation significantly inhibited the proliferation and metastasis of lung adenocarcinoma (10). In this study, RNA-seq analysis of A549 cells after irradiation with 5.16 Gy of carbon ions showed that the expression of the cytosolic iron chaperone PCBP1 was significantly reduced by carbon ion radiation. PCBP1 is a multifunctional RNA-binding protein that serves as a cytoplasmic iron chaperone, binding and transferring iron to receptor proteins in mammalian cells. Studies have shown that hepatocytes deficient in PCBP1 exhibit increased production of unstable iron and reactive oxygen species, are sensitized to iron and pro-oxidants, and accumulate oxidatively damaged lipids due to the reactivity of unaccompanied iron (11). And that abnormally spliced genes following changes in PCBP1 expression levels are associated with metabolic and apoptotic processes and may be involved in metastasis (12). PCBP1 is responsible for iron transport during various cellular life processes, and its role in regulating lung adenocarcinoma cell death in response to carbon ion radiation is unknown.

Ferroptosis is an iron-dependent mode of cell death driven by the excessive accumulation of lipid peroxides in cell membranes. ACSL4 promotes ferroptosis by facilitating the esterification of PUFAs to acyl-coenzyme A (acyl-CoA). Ionizing Radiation (IR) induces elevated ROS and ACSL4 expression in cancer cells, promoting ferroptosis (13). Lipid peroxidation promoting ferroptosis occurs when PUFAs are oxidized through the ALOX15 as well as LPCAT3 and ACSL4 pathways (14). Upregulation of ACSL4 is a hallmark of ferroptosis.PCBP1 and the heme oxygenase HO-1 are involved in the regulation of ferroptosis (15). The critical role of PCBP1 in the carbon ion-induced ferroptosis process was verified using siRNA technology in this study. Carbon ions promoted an increase in free Fe^2+^ levels in lung adenocarcinoma cells by down-regulating PCBP1 expression in NSCLC cells. ALOX15 is a non-heme iron-containing protein known for the production of PUFAs (16). It has been shown that depletion of PCBP1 leads to iron accumulation and inhibits tumor growth by blocking p62 degradation (17). ALOX15 catalyzes the stereotactic oxygenation of PUFAs, such as arachidonic acid and linoleic acid, which leads to lipid peroxidation and further induces a ferroptosis response (18). Iron levels in cells are regulated by molecules related to iron homeostasis (19). In this study, big data analysis revealed that PCBP1, ACSL4, and ALOX15 were differentially expressed in both tumor and normal tissues of NSCLC. Furthermore, high expression of all three was associated with a survival benefit in NSCLC patients. Studies have shown that down-regulation of ALOX15 during tumorigenesis may enhance the association between colitis and colorectal tumorigenesis (20), and ALOX15 is able to significantly promote ferroptosis and inhibit proliferation of gastric cancer cells (21).

It has been found that ALOX15 can regulate the sensitivity of colorectal cancer cells to ionizing radiation, which can help patients overcome tumor radiation resistance (22). We observed a significant increase in lipid peroxidation in lung adenocarcinoma cells after carbon ion radiation. Additionally, studies have shown that the intestine and colon demonstrated higher levels of the lipid peroxidation product, 4-hydroxynonenal (4-HNE), following carbon ion radiation (23), and that knockdown of PCBP1 promotes PUFA production and lipid peroxidation through activation of fatty acid synthesis and ALOX15 expression. In this study, carbon ions regulate the process of ferroptosis by inducing PCBP1 expression. The main molecular mechanism involves promoting ACSL4 expression, which induces lipid peroxidation injury by increasing Fe^2+^ transport. The key molecule of ACSL4 in ferroptosis primarily involves PUFA-PLs that are integral to lipid metabolism (24). We also found that carbon ion radiation induced ROS and lipid peroxidation, together with ferrous ions transported by PCBP1, promoted ferroptosis in NSCLC cells. Knockdown of PCBP1 increased the unstable iron pool. The accumulation of ferrous iron promotes mitochondrial dysfunction, while ACSL4 and ALOX15 accelerate the oxidation of PUFA, leading to lipid peroxidation and the occurrence of ferroptosis. This study establishes a critical role for ferroptosis in carbon ion radiation therapy and further demonstrates PCBP1 role in mediating ferroptosis in response to ionizing radiation to inhibit lung adenocarcinoma proliferation. PCBP1 expression levels in tumors may serve as predictors for prognostic assessment and response to cancer therapy involving ferroptosis inducers. Targeting PCBP1 may represent a potential therapeutic strategy to sensitize resistant cells to irradiation by promoting ferroptosis susceptibility.

## Data Availability

The original contributions presented in the study are included in the article/Supplementary material, further inquiries can be directed to the corresponding authors.

## Footnotes

^1^ https://xenabrowser.net/

^2^ https://www.proteinatlas.org/

^3^ http://kmplot.com
