# Supplementary figures and images for: The role of PCBP1 in carbon ion-induced ferroptosis and inhibition of lung adenocarcinoma proliferation
Source: Front Public Health. 2025 Jan 8;12:1496439. doi: 10.3389/fpubh.2024.1496439 (PMC11750667; doi:10.3389/fpubh.2024.1496439)

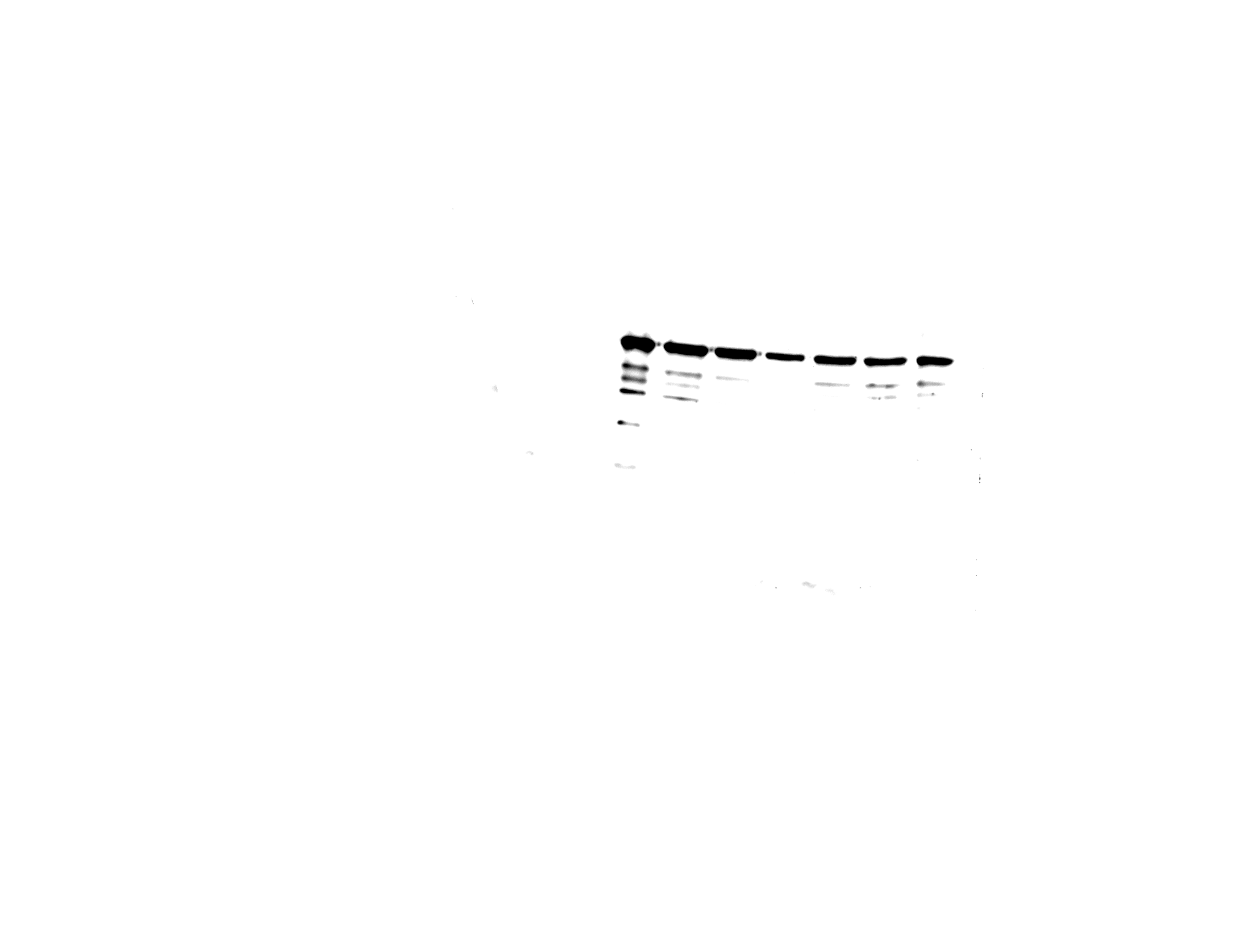

Supplement: Supplementary file 1 [file Image_1.TIF]

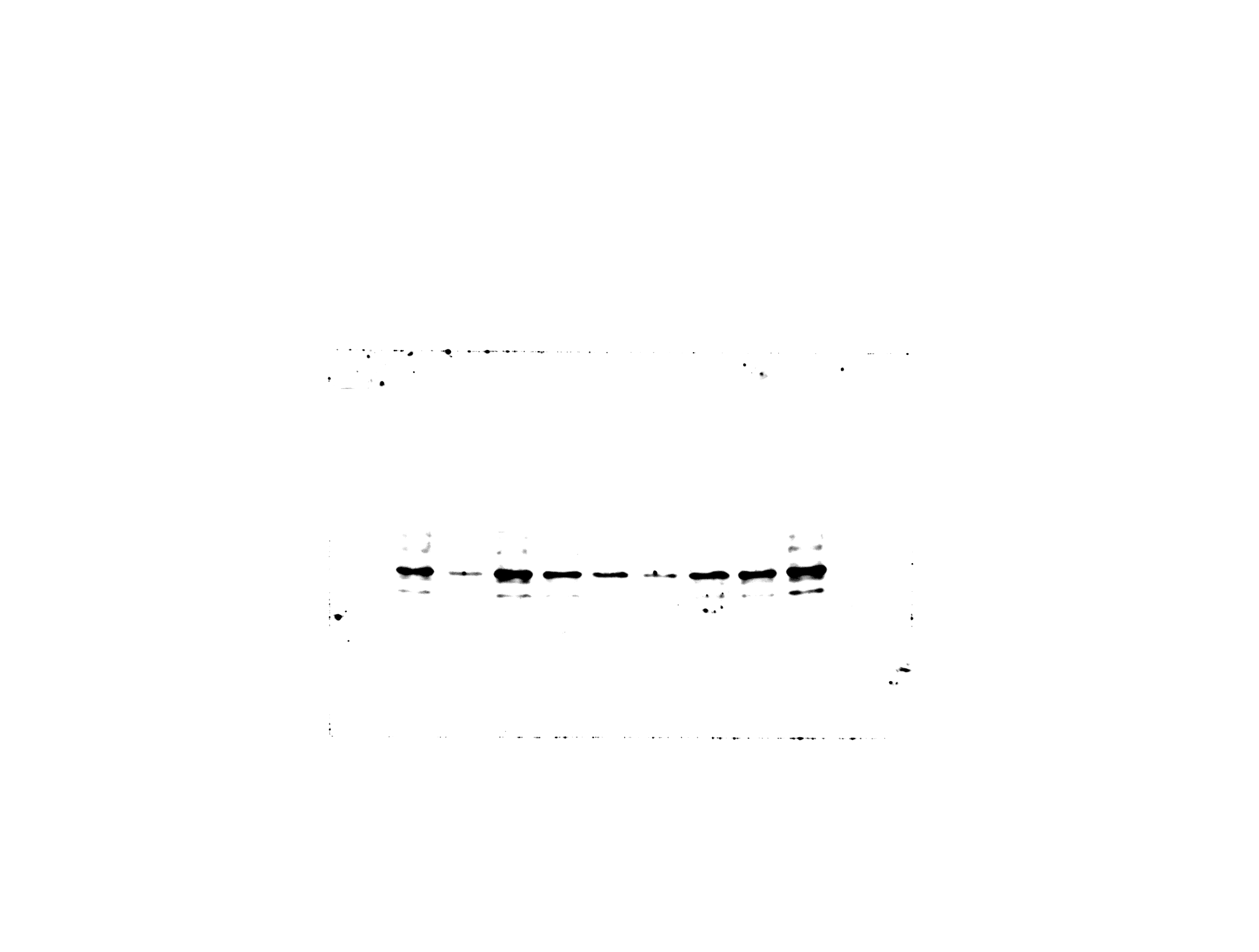

Supplement: Supplementary file 2 [file Image_2.TIF]

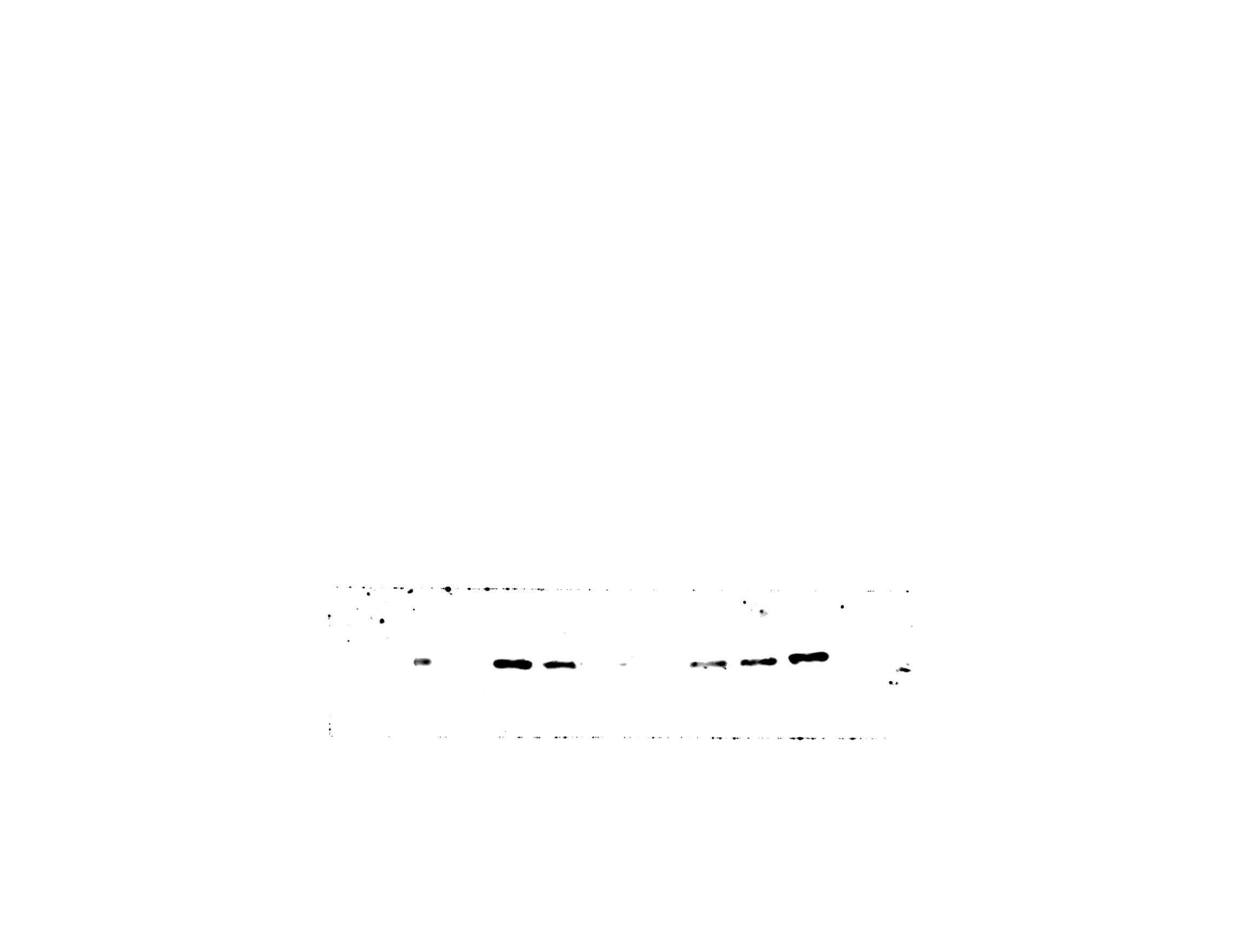

Supplement: Supplementary file 3 [file Image_3.TIF]

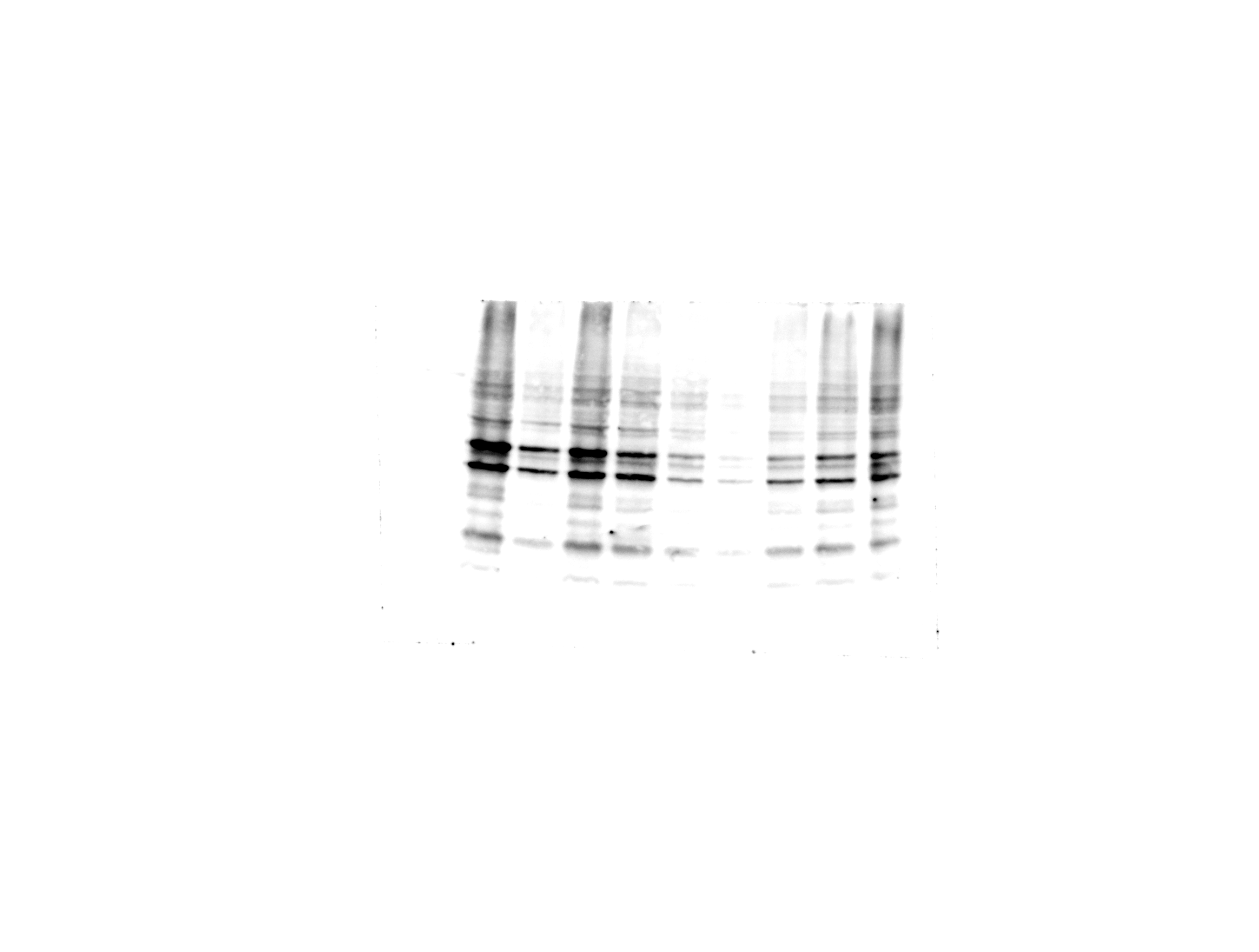

Supplement: Supplementary file 4 [file Image_4.TIF]
